# Antioxidant, Enzyme, and H_2_O_2_-Triggered Melanoma Targeted Mesoporous Organo-Silica Nanocomposites for Synergistic Cancer Therapy

**DOI:** 10.3390/antiox11112137

**Published:** 2022-10-28

**Authors:** Hyung Woo Choi, Jae Hyun Lim, Taewook Kang, Bong Geun Chung

**Affiliations:** 1 Department of Mechanical Engineering, Sogang University, Seoul 04107, Korea; 2 Department of Biomedical Engineering, Sogang University, Seoul 04107, Korea; 3 Department of Chemical and Biomolecular Engineering, Sogang University, Seoul 04107, Korea; 4 Institute of Integrated Biotechnology, Sogang University, Seoul 04107, Korea

**Keywords:** glutathione, hyaluronidase, hydrogen peroxide, mesoporous organo-silica, melanoma cells, chemo-photothermal, photodynamic therapy

## Abstract

The multi-stimuli responsive drug delivery system has recently attracted attention in cancer treatments, since it can reduce several side effects and enhance cancer therapeutic efficacy. Herein, we present the intracellular antioxidant (glutathione, GSH), enzyme (hyaluronidase, HAase), and hydrogen peroxide (H_2_O_2_) triggered mesoporous organo-silica (MOS) nanocomposites for multi-modal treatments via chemo-, photothermal, and photodynamic cancer therapies. A MOS nanoparticle was synthesized by two-types of precursors, tetraethyl orthosilicate (TEOS) and bis[3-(triethoxysilyl)propyl] tetrasulfide (BTES), providing large-sized mesopores and disulfide bonds cleavable by GSH. Additionally, we introduced a new *β*-cyclodextrin-hyaluronic acid (CDHA) gatekeeper system, enabling nanocomposites to form the specific interaction with the ferrocene (Fc) molecule, control the drug release by the HAase and H_2_O_2_ environment, as well as provide the targeting ability against the CD44-overexpressing melanoma (B16F10) cells. Indocyanine green (ICG) and doxorubicin (Dox) were loaded in the MOS-Fc-CDHA (ID@MOS-Fc-CDHA) nanocomposites, allowing for hyperthermia and cytotoxic reactive oxygen species (ROS) under an 808 nm NIR laser irradiation. Therefore, we demonstrated that the ID@MOS-Fc-CDHA nanocomposites were internalized to the B16F10 cells via the CD44 receptor-mediated endocytosis, showing the controlled drug release by GSH, HAase, and H_2_O_2_ to enhance the cancer therapeutic efficacy via the synergistic chemo-, photothermal, and photodynamic therapy effect.

## 1. Introduction

Chemotherapy has been known in a clinical practice and is commonly used in cancer treatment [1,2,3]. However, chemotherapy often causes a high drug resistance, serious side effects to normal tissues due to the lack of targeting ability, and a low permeability to cancer cells [1,2,3,4,5]. Although the biomaterial-based drug delivery system (DDS) has a great potential to solve these limitations, it requires the accurate delivery to target cancer cells, a high loading capacity of drug molecules, and a high therapeutic efficacy [6,7]. To avoid drug leakage before reaching the cancer cells and cause serious toxicity to normal tissues, the cancer targeted stimuli-responsive nanocarriers have widely been exploited in various cancer treatments [8,9,10]. The single stimuli-responsive DDS exhibits an improved cellular uptake, high accumulation rates in cancer cells, and a controlled drug release in response to the specific internal or external stimuli, including the pH, redox potential, temperature, biomolecules, light, and magnetic field [10,11,12,13]. In addition, two or more stimuli-responsive nanocarriers not only include all of the advantages in a single responsive DDS, but also accelerate the drug release at the target site, leading to several-fold increased cancer therapeutic efficiency [14,15,16].

Recently, a number of studies have focused on the mesoporous silica nanoparticle (MSN)-based nanocomposite which has excellent advantages (e.g., a high biocompatibility, a large surface area and pore volume, a uniform shape, and a facile surface modification) for stimuli-responsive and multi-modal combination therapy [17,18,19,20]. However, the relatively small pore size (~3 nm) in the MSN-based nanocomposite makes it difficult to utilize for effective cancer treatment. To address this limitation, the large pore mesoporous organo-silica (MOS) with an organic–inorganic hybrid framework has emerged as an alternative nanocarrier. The large pore-sized MOS can be achieved by co-condensation with organo-silane precursors (R-Si-(OEt)_3_, R = organic groups) [17,21,22]. In particular, the MOS nanoparticles with bis[3-(triethoxysily)propyl] tetrasulfide (BTES) molecules have been developed for stimuli-responsive DDS applications, because the tetrasulfide chain in BTES affects the formation of enlarged pores in nanoparticles and can be cleaved by an intracellular antioxidant, such as glutathione (GSH) [23,24]. To reduce the serious side effects caused by drug leakage and frequent administration, and to improve the therapeutic efficacy against cancer cells, a MOS-BTES nanocarrier needs to include all of the aforementioned strategies: (1) conjugation of the cancer-specific ligands (e.g., aptamers, antibodies, and overexpressed receptors), (2) induction of the accelerated drug release by two or more stimuli, (3) co-encapsulation of chemical drugs and NIR-mediated dual photoagents inside large pore MOS-BTES for the synergistic combination therapy [25,26,27,28,29]. Li et al. synthesized the corrosion inhibitor molecule loaded hollow MOS-BTES nanoparticles and demonstrated the pH and redox double stimuli-responsive DDS [30]. The large amount of inhibitor molecules was stably loaded in large pores (3.79 nm) and exhibited the controlled release by the pH and redox dual-response. However, this system was not suitable for use in cancer therapy due to the large particle size (about 500 nm) and low cell-internalization rates. Cheng et al. constructed dual-responsive mesoporous silica-coated gold nanorods for triple-combination therapy [31]. Since the intracellular GSH and enzyme degradable MOS silica was formed in a thin layer of gold nanorods, they showed relatively low loading capacities of doxorubicin (Dox) (5%) and the photothermal agent (3.5%), respectively. Although a number of studies, based on large pore MOS-BTES have been conducted, it has not been explored in terms of the multi-stimuli responsive nanocomposites with a three multi-modal cancer therapy.

Here, we developed a MOS-BTES based nanocomposite with a gatekeeper system that can induce selective endocytosis to melanoma cells and regulate the intracellular biomolecules-triggered drug release. Furthermore, an indocyanine green (ICG), which was capable of an elevated temperature for photothermal therapy and the generation of reactive oxygen species (ROS) for photodynamic therapy, under an 808 nm NIR laser irradiation, and Dox were co-encapsulated in the nanocomposites, resulting in the synergistic multi-modal cancer therapy. A gatekeeper was designed on the fact that hyaluronic acid was degraded by the intracellular hyaluronidase (HAase) enzyme, selectively internalized in the CD44-overexpressing melanoma cells, and the guest–host interactions between ferrocene (Fc) and β-cyclodextrin (β-CD), were significantly affected by the hydrogen peroxide (H_2_O_2_) environment in cancer cells [20,32,33]. The large pore MOS was modified by Fc molecules, and the β-cyclodextrin-hyaluronic acid (CDHA) gatekeeper was conjugated, subsequently. We observed that the MOS-Fc-CDHA nanocomposites exhibited no cytotoxicity against the normal NIH-3T3 and melanoma B16F10 cells and the selective cellular uptake in the B16F10 cells via the CD44-receptor mediated endocytosis pathway. Furthermore, the MOS-Fc-CDHA nanocomposites showed the excellent controlled drug release by the intracellular GSH, HAase, H_2_O_2_ condition as well as exhibited a synergistic therapeutic effect of the melanoma cells via chemo-, photothermal, and photodynamic therapies.

## 2. Materials and Methods

### 2.1. Materials

Tetraethylorthosilicate (TEOS), 3-aminopropyltriethoxysilane (APTES), cetyltrimethylammonium chloride solution (CTAC, 25 wt%), triethanolamine (TEA), glutathione (GSH), toluenesulfonyl chloride (TsCl), ethylenediamine, 1-ethyl-3-(3-dimethylaminopropyl) carbodiimide (EDC), N-hydroxysuccinimde (NHS), and hyaluronidase from bovine testes (HAase, 400–1000 units/mg) were purchased from Sigma-Aldrich (St. Louis, MO, USA). BTES was obtained from Gelast, Inc. (Morrisville, PA, USA). 1,3-diphenylisobenzofuran (DPBF), *β*-cyclodextrin (*β*-CD), indocyanine green (ICG), doxorubicin hydrochloride (Dox), and *N*-succinimidyl ferrocenecarboxylate (Fc-NHS) were purchased from Tokyo Chemical Industry Co. Ltd. (Tokyo, Japan). Hyaluronic acid (5 kDa, HA) was obtained from Lifecore Biomedical, Inc. (Chaska, MN, USA). A 2′,7′-dichlorofluorescein diacetate (DCFDA) assay kit for the detection of cellular ROS was obtained from Abcam, Inc. (Cambridge, MA, USA). Mouse fibroblast (NIH-3T3) and melanoma (B16F10) cells were provided from Korea Cell Line Bank (Seoul, Korea). Dulbecco`s phosphate-buffered saline (DPBS), Dulbecco`s modified Eagle`s medium (DMEM), fetal bovine serum (FBS), 4′,6-diamidino-2-phenylindole (DAPI), antibiotics, and live/dead kit were purchased from Thermo Fisher Scientific Inc. (Waltham, MA, USA). The cell proliferation kit (3-(4,5-dimethylthiozol-2-yl)-2,5-diphenyltetrazolium bromide, MTT) was obtained from Roche Diagnostics (Mannheim, Germany). Other reagents and solvents were used without further purification.

### 2.2. Synthesis of the MOS Nanoparticles

MOS nanoparticles were synthesized, according to a previously reported method with a minor modification [24]. Two mL of CTAC and 53 μL of TEA were added in 20 mL deionized water (DW) with vigorous stirring, followed by the addition of the silica precursors (1.06 mL of TEOS and 1.2 mL of BTES) at 95 °C for 4 h. Once cooled to room temperature, a MOS nanoparticle was collected by centrifugation (12,000 rpm, 10 min) and was rinsed with EtOH and DW, repeatedly. The template of the MOS was removed by treatment with 1 wt% NaCl solution (at 50 °C for 3 h). The MOS nanoparticle was washed three times with MeOH and dried at 80 °C.

### 2.3. Surface Modification of the Ferrocene Molecules on the MOS Nanoparticle

Prior to the functionalization of Fc, the surface of the MOS was changed to the amine groups [9]. Five hundred μL of APTES was slowly dropped into the MOS solution (1 mg/mL, 50 mL) and was stirred at 85 °C for 12 h. The aminated MOS (MOS-NH_2_) nanoparticle was centrifuged with EtOH and DW (12,000 rpm, 10 min). Subsequently, the process of the Fc molecule modified MOS (MOS-Fc) was performed following the literature [34]. MOS-NH_2_ (10 mg) and Fc-NHS (10 mg) dissolved in PBS (pH 5.7) were sonicated for 30 min and were vigorously stirred for 18 h. The MOS-Fc and the unreacted chemicals were isolated by centrifugation and were purified several times with MeOH. The obtained MOS-Fc nanoparticle was dried at 80 °C for further use.

### 2.4. Synthesis of the Cancer Targeted and Stimuli-Responsive CDHA Gatekeeper

The melanoma targeted and stimuli-responsive β-cyclodextrin-hyaluronic acid (CDHA) gatekeeper was synthesized in three steps [35]. First, 4.0 g of β-CD and 3.0 g of TsCl were dissolved in NaOH solution (0.5 M, 50 mL) and the mixture was stirred at 0 °C for 1 h. Once the reaction was completed, the reactant was filtered and tosylated. β-CD (Ts-β-CD) was neutralized by using 0.1 M HCl solution. Second, Ts-β-CD (3.0 mg) was reacted with ethylenediamine (20 mL) at 40 °C for 24 h. The purification of the aminated β-CD (*β*-CD-NH_2_) was carried out by a precipitation with acetone. Finally, EDC (0.52 mmol) and NHS (0.87 mmol) were added sequentially to the HA solution (10 mL, 5 mg/mL, pH 5.8) and were sonicated for 30 min. *β*-CD-NH_2_ (30 mg) was mixed into the above solution and was maintained for 18 h. The melanoma targeted and stimuli-responsive CDHA gatekeeper was purified by a dialysis membrane (molecular weight cut off: 6–8 kDa) against DW for two days and was acquired by freeze-drying.

### 2.5. Preparation of the Intracellular Antioxidant, Enzyme and H_2_O_2_ Triggered MOS-Fc-CDHA Nanocomposite for the Combined Chemo-, Photothermal and Photodynamic Therapy

To enhance the therapeutic efficacy against the melanoma cells, the NIR-mediated photoagent (ICG) and Dox were co-loaded into the MOS-Fc nanoparticle [36]. ICG (5 mg) and Dox (5 mg) was added to the MOS-Fc nanoparticle solution (10 mL) and were stirred for 24 h. The ICG and Dox encapsulated MOS-Fc (ID@MOS-Fc) nanoparticle was collected by ultra-centrifugation (14,000 rpm, 10 min) and was freeze-dried for 48 h. Then, the cancer targeted and stimuli-responsive CDHA gatekeeper was conjugated on the MOS-Fc nanoparticle by the specific binding interaction between Fc and *β*-CD [32]. The ID@MOS-Fc and CDHA gatekeeper were mixed in the same ratio (1:1) and were stirred at room temperature for 24 h. Following the washing with DW, the ICG-Dox co-loaded MOS-Fc-CDHA nanocomposite (ID@MOS-Fc-CDHA) was obtained by lyophilization. The presence of ICG and Dox in the nanocomposite was confirmed by measuring the UV-vis spectroscopy (UV 1800, Shimazu, Kyoto, Japan) at 480 nm and 780 nm and the loading efficiency and capacity were determined via conventional equations [37].

### 2.6. Characterization of the MOS-Fc-CDHA Nanocomposites

The particle size and shape of the MOS-Fc-CDHA nanocomposites were observed by transmission electron microscopy (TEM, JEOL-2100, Tokyo, Japan). High-resolution TEM images and elemental mapping of the nanocomposite were obtained by energy dispersive X-ray spectroscopy (EDX). The surface charge was determined, using a Zetasizer Nano ZS90 (Malvern Instruments, Malvern, UK) and the particle size and distribution were measured by ELS-1000ZS (Otsuka Electronics, Osaka, Japan). To confirm the change of the average pore size and the surface area by the CDHA gatekeeper, Brunauer–Emmett–Teller (BET) and Barret–Joyner–Halenda (BJH) were performed by nitrogen gas adsorption-desorption isotherms using an automatic adsorption apparatus, ASAP analyzer (Micromeritics, Inc., Narcross, GA, USA). The amount of the grafted Fc and CDHA on the MOS nanoparticles was determined using a Discovery TGA (TA Instruments, New Castle, DE, USA). Fourier infrared spectroscopy (FT-IR, Vertex 70, Bruker Inc., Billerica, MA, USA) was used for the chemical structure of the MOS-Fc-CDHA nanocomposites. The synthesized CDHA gatekeeper was evaluated by ^1^H nuclear magnetic resonance spectroscopy (NMR, UNITY-Inova 500, Palo Alto, CA, USA) and the FT-IR spectra were recorded on a Nicolet iS 50 (Thermo Fisher Scientific Inc., Waltham, MA, USA). The ICG and Dox in the MOS-Fc-CDHA nanocomposites were verified using a UV-vis spectroscopy.

### 2.7. Photothermal and Photodynamic Performances of the ID@MOS-Fc-CDHA Nanocomposites under NIR Laser Irradiation

The NIR laser-mediated phototherapeutic potential of the ID@MOS-Fc-CDHA nanocomposites was investigated before the in vitro test, using the melanoma cells. First, the photothermal effect was evaluated by real-time temperature recording under the NIR laser irradiation (BWF2, B&W Tek, Inc., Newark, DE, USA). We prepared various nanocomposite solutions (0.05–0.2 mg/mL) and exposed them to the 808 nm NIR laser for 10 min at a power density of 1 W/cm^2^. The effect of the laser power density was monitored by increasing the temperature of the nanocomposite solution irradiated with different power densities (0.5–2.0 W/cm^2^). To investigate the photothermal stability via NIR light, the ID@MOS-Fc-CDHA solution was irradiated with an 808 nm NIR laser for 10 min, and the following natural cooling process was performed three times. The temperature changes of the nanocomposite were recorded by a thermo-coupler connected to a digital thermometer (DTM-318, TECPEL Co., Ltd., New Taipei, Taiwan). Furthermore, we demonstrated the photodynamic property of the ID@MOS-Fc-CDHA nanocomposites through the ROS generation by the NIR laser. The ROS generated from the ID@MOS-Fc-CDHA nanocomposites was confirmed using a DPBF probe that could react specifically with the ROS [38]. In detail, 10 μL DPBF solution (1 mg/mL, DW) was added to 100 μL ID@MOS-Fc-CDHA (0.1 mg/mL) and were mixed in a dark space. The mixture was treated with an 808 nm NIR laser (1 W/cm^2^) every 2 min for 10 min. As a control, the DPBF solution was performed in the same manner. The absorption curves of the ROS generation were obtained by UV-vis spectroscopy (410 nm).

### 2.8. Antioxidant, Enzyme, and H_2_O_2_ Responsive Dox Release from the ID@MOS-Fc-CDHA Nanocomposites

The intracellular antioxidant, enzyme, and H_2_O_2_ responsive Dox release from the nanocomposites was conducted in four different conditions. ID@MOS-Fc-CDHA (1 mg/mL in PBS) was treated with GSH (3.2 mM), HAase (1 mg), H_2_O_2_ solution (1% *v*/*v*), and GSH-HAase-H_2_O_2_ mixture, respectively. These solutions were incubated at 37 °C for 12 h. Following the pre-determined time intervals, an aliquot (0.1 mL) was carefully withdrawn from each solution, followed by ultra-centrifugation (14,000 rpm, 10 min). The cumulative release profile of the ID@MOS-Fc-CDHA nanocomposites was obtained by measuring the UV-vis spectroscopy at 480 nm. In addition, the changes of the ID@MOS-Fc-CDHA nanocomposites treated with the intracellular GSH, HAase, and H_2_O_2,_ was observed by the high-resolution TEM.

### 2.9. Cytotoxicity Analysis of the ID@MOS-Fc-CDHA Nanocomposites

The cell viability test of the MOS-Fc-CDHA nanocomposites was performed by a MTT assay. Briefly, normal (NIH-3T3) and melanoma (B16F10) cells were seeded in 96-well plates, at a density of 1 × 10^4^ cells per well and incubated for 24 h at 37 °C, under a humidified atmosphere with 5% CO_2_. One hundred μL of the ID@MOS-Fc-CDHA nanocomposites with various concentrations (10–100 μg/mL) was treated in each well, and the 96-well plate was incubated again for 24 h. The cells were washed with DPBS, and the fresh medium containing MTT agent (0.5 mg/mL) was added and were incubated for 4 h. The medium was carefully removed and 200 μL of DMSO was added. The cell viabilities of the NIH-3T3 and B16F10 cells against the ID@MOS-Fc-CDHA nanocomposites were quantified using an iMark^TM^ microplate reader (Bio-rad, Hercules, CA, USA).

### 2.10. Melanoma Targeted Cellular Uptake of the ID@MOS-Fc-CDHA Nanocomposites

The selective cellular uptake of the ID@MOS-Fc-CDHA nanocomposites in the melanoma cells was evaluated using confocal laser scanning microscopy (CLSM, LSM 880, Carl Zeiss, Jena, Germany). The NIH-3T3 and B16F10 cells (2 × 10^4^ cells/well) were seeded in 8-well plates (ibidi, Munich, Germany) and were incubated with the ID@MOS-Fc-CDHA nanocomposites (100 μg/mL) for 6 h. The non-internalized nanocomposite was removed by PBS washing three times and then was fixed with 4% paraformaldehyde for 15 min. Following the treatment with Triton X-100 (0.1% *v*/*v*) and BSA (2 wt%) for 10 min, the cells were stained with 4,6-diamidino-2-phenylindole (DAPI, Thermo Fisher Scientific, Waltham, MA, USA) for 5 min.

### 2.11. Detection of the Intracellular ROS Generated by the ID@MOS-Fc-CDHA Nanocomposites

The ROS generation in the B16F10 cells treated with the ID@MOS-Fc-CDHA nanocomposites was confirmed by the DCFDA analysis [39]. The B16F10 cells were cultured in a 96-well plate and were incubated with the ID@MOS-Fc-CDHA (60 μg/mL) for 6 h. To evaluate the intracellular ROS generation, the cells were exposed to an 808 nm NIR laser (2 W/cm^2^, 5 min) and then washed several times with DPBS. DCFDA (50 μM) was added to each well and was incubated for 40 min. The fluorescence signal inside the melanoma cells was detected using inverted fluorescence microscopy (Olympus IX73, Tokyo, Japan).

### 2.12. Enhanced Therapeutic Efficacy of the ID@MOS-Fc-CDHA Nanocomposite via Chemo-, Photothermal, and Photodynamic Therapy

The synergistic anti-cancer effect of the ID@MOS-Fc-CDHA nanocomposites via the multi-modality toward the melanoma cells was evaluated by a MTT assay. The B16F10 cells were seeded in a 96-well plate at a density of 1 × 10^4^ cells per well. Twenty-four h later, the ID@MOS-Fc-CDHA nanocomposites with various concentrations (20–60 μg/mL) were added to the melanoma cells and stored at 37 °C for 6 h. To evaluate the therapeutic effect via the chemo-, photothermal, and photodynamic therapies, the B16F10 cells with the ID@MOS-Fc-CDHA nanocomposites were irradiated with an 808 nm NIR laser (2 W/cm^2^, 5 min). As control groups, the B16F10 cells were treated with ID@MOS-Fc-CDHA without the NIR irradiation (chemotherapy) and the B16F10 cells with ID@MOS-Fc-CDHA were incubated in the DMEM medium, including the NaN_3_ ROS inhibitor (10 μM) and were irradiated by the NIR laser irradiation (chemo- and photothermal therapies) [40]. The B16F10 cells were washed with DPBS and the MTT assay was performed in the same manner as in the 2.9 cytotoxicity analysis method. Furthermore, to visualize the therapeutic effect by the ID@MOS-Fc-CDHA nanocomposites, a live/dead assay was carried out. ID@MOS-Fc-CDHA (60 μg/mL) was treated into B16F10 for 24 h and then were exposed with or without the NIR laser irradiation (2 W/cm^2^, 5 min). For the chemo- and photothermal therapies, the B16F10 cells with ID@MON-Fc-CDHA were incubated in the DMEM medium, including NaN_3,_ and were irradiated by the NIR laser. These cells were stained with calcein AM and ethidium homodimer-1 and were washed with DPBS. The live/dead images were acquired with an Olympus IX73 microscopy.

## 3. Results and Discussions

### 3.1. Synthesis and Characterization of the MOS-Fc-CDHA Nanocomposites

**Scheme 1:**
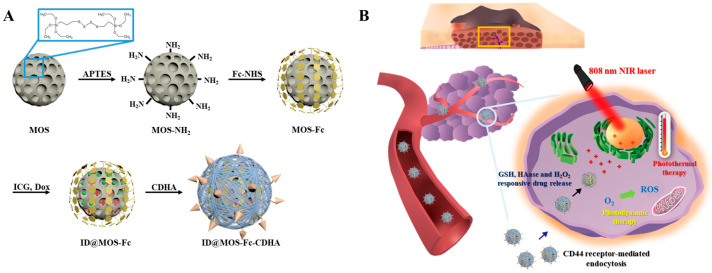
(**A**) Synthesis process of the multi-triggered melanoma targeted ICG-Dox co-loaded MOS-Fc-CDHA nanocomposite and (**B**) Illustration of the GSH, HAase and H_2_O_2_ responsive drug release and synergistic chemo-, photothermal, and photodynamic therapies in melanoma cells.

The MOS nanoparticle with a large pore structure and redox cleavable behavior by the intracellular GSH in the melanoma cells was synthesized through the hydrolysis and condensation reaction of the silica precursor (TEOS) and the silica co-precursor (BTES) with thioether-bridged groups [24]. To provide oxidant sensitivity in the nanoparticles, we adopted the host-guest interaction between the Fc molecule and *β*-CD, which could be cleaved by H_2_O_2_ [32]. Following the surface modification to the Fc groups, ICG and Dox, as therapeutic agents, were co-encapsulated into the large pore MOS-Fc via a hydrophobic interaction. Finally, by conjugation of the CD-HA gatekeeper, our nanocomposite showed the specific cancer targeting ability to the CD44-overexpressing melanoma cells, the controlled Dox release by the intracellular antioxidant and enzyme (GSH and HAase), and the enhanced therapeutic effects by the chemo-, photothermal, and photodynamic therapies (Scheme 1).

**Figure 1:**
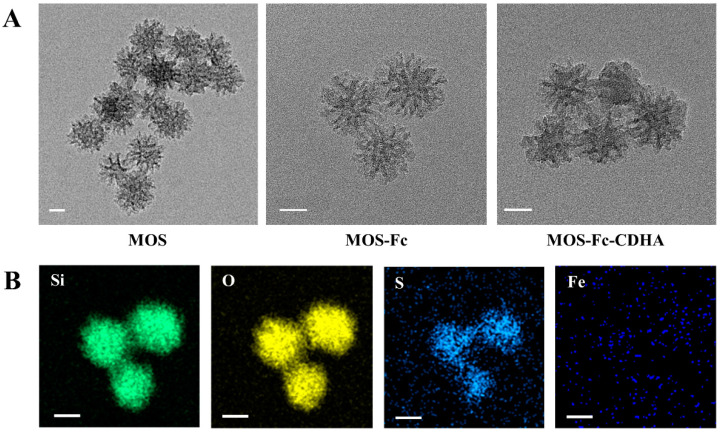
(**A**) TEM images of the MOS, MOS-Fc, and MOS-Fc-CDHA nanocomposites. (**B**) EDX mapping images of the MOS-Fc-CDHA nanocomposites. Scale bars are 20 nm.

Figure 1 shows the size, morphology, and elemental composition of the MOS, MOS-Fc, and MON-Fc-CDHA nanocomposites. In Figure 1A, as compared to the mesoporous silica (MS) with small pores, the large pore MOS nanoparticle exhibited the well-defined and monodispersed urchin-like shape with an average diameter of 42 ± 1.29 nm, due to the hydrophobic tetrasulfide chains (-CH_2_-S-S-S-S-CH_2_-) in the BTES molecule [23]. Following the modification with the Fc group and the conjugation with the CDHA gatekeeper, the morphology of the original MOS nanoparticles was maintained, however, the sizes of the MOS-Fc and MOS-Fc-CDHA slightly increased to 46.6 ± 3.54 and 48.6 ± 1.14 nm. Moreover, the hydrodynamic diameters of the MOS, MOS-Fc, MOS-Fc-CDHA also increased (77.8, 99.9, and 126.6 nm) and these changes were corresponded with the TEM (Figure S1). In Figure 1B, the elemental composition of the MOS-Fc-CDHA nanocomposite was analyzed by EDX mapping, and the main elements of the nanocomposite were Si, O, S, and Fe. In particular, the S and Fe elements were approximately 7% and 2.5%, respectively, due to the tetrasulfide chain and Fc group in the nanocomposites.

**Figure 2:**
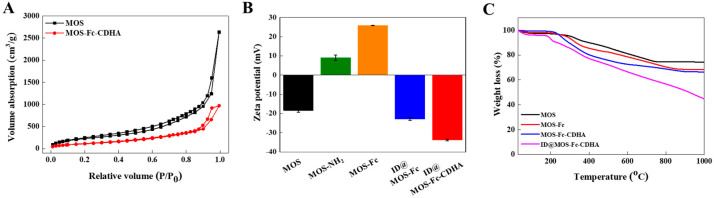
Characterization of the ID@MOS-Fc-CDHA nanocomposites. (**A**) N_2_ adsorption/desorption isotherm of the MOS and MOS-Fc-CDHA nanocomposites. (**B**) Zeta-potential of the MOS, MOS-NH_2_, MOS-Fc, ID@MOS-Fc, and ID@MOS-Fc-CDHA nanocomposites. (**C**) TGA analysis of the MOS, MOS-Fc, MOS-Fc-CDHA, and ID@MOS-Fc-CDHA nanocomposites.

The N_2_ sorption isotherm of the MOS and MOS-Fc-CDHA nanocomposites was displayed in Figure 2A. The MOS nanoparticle was composed of well-defined porous structures with a large surface area of 919.15 m^2^/g, a high pore volume of 4.03 cm^3^/g, and a large pore size of 5.6 nm. However, the MOS-Fc-CDHA showed a smaller surface area, pore volume, and pore size (416.7 m^2^/g, 1.5 cm^3^/g, and 1.4 nm), because the CDHA gatekeeper was completely covered with the large pore MOS nanoparticles by the Fc-CD specific interaction. The zeta potential of the MOS-Fc-CDHA nanocomposites following the surface modification, was shown in Figure 2B. The charges of MOS, MOS-NH_2_, MOS-Fc, ID@MOS-Fc, and ID@MOS-Fc-CDHA were −18.4 ± 0.9, +9.03 ± 1.5, +25.9 ± 0.1, −22.9 ± 0.751, and −33.8 ± 0.416 mV, respectively. These results indicated that the properties of the nanocomposites were sequentially changed, depending on the amination, the replaced Fc molecule, the co-encapsulation of ICG and Dox, and the conjugation by the CDHA gatekeeper. The amount of the Fc molecule, CDHA, and the co-loaded ICG and Dox in the nanocomposites were determined by the TGA analysis (Figure 2C). During the temperature rise up to 1000 °C, MOS-Fc, MOS-Fc-CDHA, and ID@MOS-Fc-CDHA showed a weight loss of 31.5, 33.5, and 55.4%, as compared to the MOS nanoparticles (25.5%). This result implied that 6% of Fc, 2% of the CDHA gatekeeper, and 22% of the co-loaded ICG and Dox were contained in the nanocomposites. Prior to the analysis of the FT-IR and UV-vis spectroscopy on the MOS-Fc-CDHA nanocomposites, the melanoma targeted and stimuli-responsive CDHA gatekeeper was performed using the H NMR measurement. As previously mentioned in the materials and methods section, *β*-CD was sequentially converted to Ts-*β*-CD and *β*-CD-NH_2_, and then the HA molecule was grafted using EDC/NHS chemistry (Scheme S1). Compared with *β*-CD-NH_2_, the spectrum of the CDHA gatekeeper exhibited the intrinsic peak of *β*-CD at 3.6 ppm and the new characteristic peaks of the HA molecule appeared at 2.0, 3.5, and 3.8 ppm, which verified the synthesis of the CDHA gatekeeper (Figure S2) [41,42]. The chemical bonding of the MOS, MOS-Fc, and MOS-Fc-CDHA nanocomposites was certified by the FT-IR spectra, as shown in Figure S3. The three curves showed typical vibrations at 800, 960, and 1090 cm^−1^, corresponding to the asymmetric and symmetric stretching of Si-O-Si and Si-OH [24,43]. The absorption peaks of S-C and S-S were observed at 570 and 690 cm^−1^, attributing to the GSH cleavable tetrasulfide chain (BTES) [24,43]. In the spectrum of the MOS-Fc nanoparticles, the new vibration peaks of C=O, N-H, and C=C, appeared at 1640, 1540, and 1450 cm^−1^, respectively [20,34]. The successful conjugation of the CDHA gatekeeper with the MOS-Fc nanoparticles was demonstrated by the new peaks at 1635, 2870, and 3450 cm^−1^, corresponding to the stretching vibrations of C=O (HA), -OH, and -CH (*β*-CD) [44,45]. Finally, the co-encapsulated ICG and Dox in the MOS-Fc-CDHA nanocomposites were confirmed by UV-vis spectroscopy (Figure S4). As shown in the UV spectra, the ID@MOS-Fc-CDHA presented broad absorptions at 480 and 780 nm, corresponding to the intrinsic absorption peaks of ICG and Dox [46]. The UV-vis spectrum indicated that the MOS-Fc-CDHA nanocomposites showed a great potential for the multi-modal treatment. Moreover, the loading capacity of ICG and Dox, alone was determined by UV-vis spectroscopy, showing 12.8 and 12.6%, respectively, using the previous equation [37]. The total loaded capacity of ICG and Dox in the nanocomposites obtained by this formula (25.4%), was reflected in the amount of weight loss by the TGA analysis (22%). This value was much higher than the general MS with small pores (about 10%), indicating ICG and Dox were well-encapsulated to the large pore MOS nanoparticles [37].

### 3.2. Photothermal and Photodynamic Performances of the ID@MOS-Fc-CDHA Nanocomposites via the NIR Laser Irradiation

**Figure 3:**
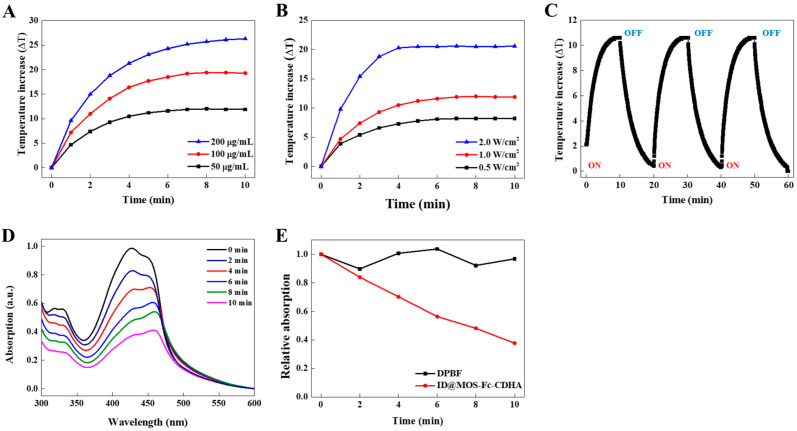
Photothermal and photodynamic performances of the ID@MOS-Fc-CDHA nanocomposites. (**A**) Temperature changes of the ID@MOS-Fc-CDHA nanocomposites with various concentrations under a 808 nm NIR laser (1 W/cm^2^). (**B**) Temperature curves of ID@MON-Fc-CDHA with different laser power densities (0.5–2.0 W/cm^2^). (**C**) Photostability of the ID@MON-Fc-CDHA (50 μg/mL) nanocomposites after three NIR laser on/off cycles of a 808 nm NIR laser irradiation (1 W/cm^2^, 10 min). (**D**) UV-vis spectra of the DPBF solution including the ID@MOS-Fc-CDHA nanocomposites after the 808 nm NIR laser irradiation at different time periods. (**E**) ROS generation curves of the DPBF probe and the ID@MOS-Fc-CDHA nanocomposites.

We demonstrated the photothermal property of the ID@MOS-Fc-CDHA nanocomposites by the NIR laser irradiation. As shown in Figure 3A, the temperature rose to 11.9 °C, even at a low concentration (50 μg/mL) during the NIR irradiation for 10 min (808 nm, 1 W/cm^2^). As the concentration increased gradually (100 and 200 μg/mL), the increased temperatures were from 19.3 °C to 26.3 °C. The temperature changes of the same concentration (50 μg/mL) exposed to the NIR laser with different outputs, were observed in Figure 3B. We observed that the temperature was proportionally elevated to the laser power. To investigate the photothermal stability, three cycles of “laser on/off” measurement were conducted. As shown in Figure 3C, the temperature constantly increased to 11 °C without any attenuation during the three cycles, indicating that the ID@MOS-Fc-CDHA nanocomposites exhibited an excellent photothermal stability. The ID@MOS-Fc-CDHA nanocomposites exhibited excellent photothermal properties and a photothermal conversion efficiency (18.6%) was determined and it corresponded to the previous reports for the photothermal therapy [47,48]. Additionally, the photodynamic therapy using the ID@MOS-Fc-CDHA nanocomposites was verified through the detection of the ROS generated by DPBF. In Figure 3D, the ID@MOS-Fc-CDHA (100 μg/mL) mixed with the DPBF solution was exposed to an 808 nm NIR laser (1 W/cm^2^) every 2 min followed by the UV-vis spectroscopy. When a DPBF probe reacted with the ROS, the DPBF probe was decomposed and o-dibenzoylbenzene was formed to induce the decreased intensity of absorption at 410 nm [38]. The absorption peaks at 410 nm gradually diminished with the NIR laser irradiation time. The relative absorption of the DPBF probe alone did not much change regardless of the NIR laser irradiation time. However, the ID@MOS-Fc-CDHA with the DPBF probe showed the continuous decrease in the relative absorption, depending on the exposure time of the NIR laser (Figure 3E). The Figure 3D,E indicated that the large amount of the ROS could be produced by the NIR-mediated ID@MOS-Fc-CDHA nanocomposites. Therefore, the NIR laser-mediated ID@MOS-Fc-CDHA has great photothermal and photodynamic properties, due to the effective and stable encapsulation of the ICG molecules with a high loading capacity in the nanocomposite.

### 3.3. Intracellular Antioxidant, Enzyme, and H_2_O_2_ Multi-Triggered Dox Release of the ID@MOS-Fc-CDHA Nanocomposites

**Figure 4:**
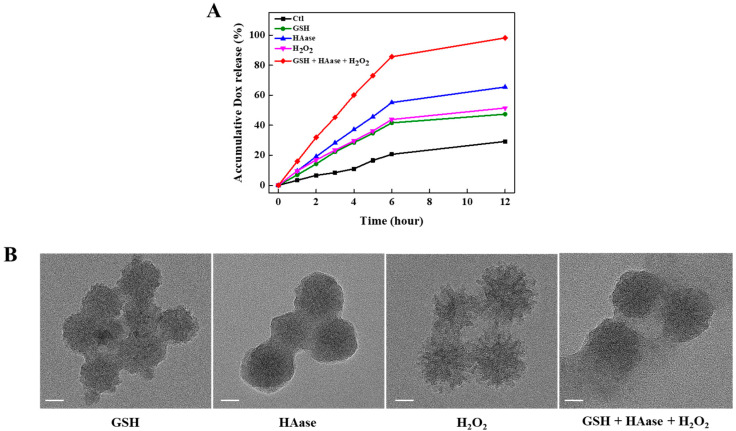
(**A**) Triple-stimuli responsive Dox release profiles from the ID@MOS-Fc-CDHA nanocomposites. (**B**) TEM images of the ID@MOS-Fc-CDHA nanocomposites treated with intracellular GSH, HAase, H_2_O_2,_ and the GSH-HAase-H_2_O_2_ mixture.

It has been known that the cancer cells show different properties to normal cells, such as high intracellular GSH levels, an oxidative microenvironment with a high concentration of H_2_O_2_, a high temperature, and a weak acidic condition [32]. In particular, the CD44-overexpressing melanoma cells contain the additional lysosomal enzyme (HAase) [49,50]. Therefore, we designed the nanocomposites with a controlled drug release by multi-triggers (e.g., intracellular GSH, lysosomal enzyme, and H_2_O_2_ environment). The multi-stimuli responsive Dox release from the ID@MOS-Fc-CDHA nanocomposites was performed for 12 h at 37 °C (Figure 4A). As a control, ID@MOS-Fc-CDHA was placed at 37 °C for 12 h without any trigger and 29.2% of the Dox release appeared. However, when ID@MOS-Fc-CDHA was treated with intracellular GSH, HAase, and the H_2_O_2_ solution, the cumulative Dox release in the nanocomposites reached to 47.4, 65.4, and 51.5%, respectively. Interestingly, in the presence of GSH, HAase, and H_2_O_2_, the faster release of Dox was observed, as compared to a single stimulus (98%). Following the treatment with various stimuli, we obtained the HR-TEM images to explain the morphology change of the ID@MOS-Fc-CDHA nanocomposites in Figure 4B. In the antioxidant treatment, the tetrasulfide chains in the nanocomposites were cleaved by the intracellular GSH, resulting in the destruction of the mesoporous silica framework and the disappearance of large pores [23]. The HAase-treated nanocomposites exhibited a closed porous structure, because only the outer HA layer on ID@MOS-Fc-CDHA was degraded by the lysosomal HAase enzyme [9,51,52]. When the nanocomposite was exposed to the H_2_O_2_ solution, the large porous structure was clearly observed, because the Fc molecule was converted to an oxidized Fc ion (Fc^+^) under the H_2_O_2_ environment, leading to the destructed host-guest interaction between the MOS-Fc and CDHA gatekeeper [32]. Therefore, in the multi-stimulus environment, the mesoporous silica framework, the outer HA layer, and the Fc-CDHA interaction were completely demolished to promote the Dox release from the ID@MOS-Fc-CDHA nanocomposites.

### 3.4. Cytotoxicity Assay and Selective Cellular Uptake of the MOS-Fc-CDHA Nanocomposites

**Figure 5:**
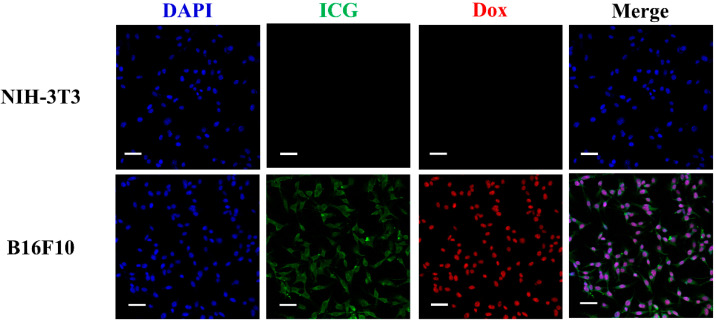
Cellular uptake behaviors of the NIH-3T3 and B16F10 cells incubated with the ID@MOS-Fc-CDHA nanocomposites. Scale bars are 20 μm. DAPI: blue, ICG: green, and Dox: red color.

The cytotoxicity analysis of the MOS-Fc-CDHA composites was evaluated using the NIH-3T3 and B16F10 cells. Figure S5 exhibited the cell viability analysis of the NIH-3T3 and B16F10 cells treated with the MOS-Fc-CDHA nanocomposites at various concentrations. The proportion of viable cells of NIH-3T3 and B16F10 remained more than 85%, even at a high concentration (100 μg/mL). This result verified that the MOS-Fc-CDHA nanocomposites showed an excellent security, biocompatibility, and negligible side effects [53]. We further confirmed the selective cellular uptake of the ID@MOS-Fc-CDHA nanocomposites against the CD44-negative cell line (NIH-3T3) and the CD44-overexpressing cell line (B16F10). Then, the ID@MOS-Fc-CDHA nanocomposites were incubated with the NIH-3T3 and B16F10 cells, the fluorescent images were observed. The cell nuclei of both cells were stained in blue (DAPI) and the subcellular localized ICG and Dox were marked with green and red (Figure 5). In the CD44-overexpressing B16F10 cells with ID@MOS-Fc-CDHA, the relatively strong green and red fluorescence were distributed in the cytoplasm and cell nucleus, respectively. However, the NIH-3T3 cells with the ID@MOS-Fc-CDHA nanocomposites exhibited no fluorescence of ICG and Dox. This result suggested that the MOS-Fc-CDHA nanocomposites were selectively internalized into melanoma cells via the CD44 receptor-mediated endocytosis, as well as the release of ICG and Dox could be controlled by the intracellular GSH, HAase, and H_2_O_2_ [54,55].

### 3.5. Intracellular ROS Generated by the ID@MOS-Fc-CDHA Nanocomposites

**Figure 6:**
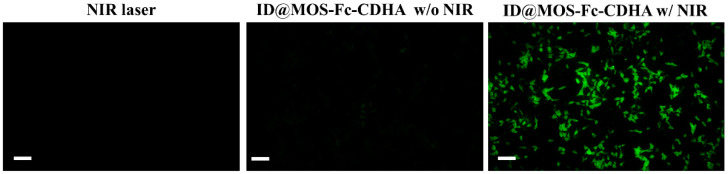
Detection of the intracellular ROS in the B16F10 melanoma cells treated with the ID@MOS-Fc-CDHA nanocomposites with (w/) or without (w/o) the NIR laser irradiation. Scale bars are 50 μm.

The generation of the ROS by the NIR laser irradiation causes the oxidative stress in cancer cells and induces the immunogenic cell death [56]. To observe the intracellular ROS in the melanoma cells, the ID@MOS-Fc-CDHA nanocomposites and the DCFDA probes were treated in the B16F10 cells and were subsequently exposed to a 808 nm NIR laser [57]. As expected, the fluorescence intensity was obviously different with the NIR laser irradiation in the melanoma cells (Figure 6). When the B16F10 cells treated with the ID@MOS-Fc-CDHA nanocomposites were not exposed to the NIR laser, the green signal was very weak, indicating no generation of the ROS in the B16F10 cells. However, the fluorescence intensity was the highest for the cells treated with ID@MOS-Fc-CDHA and the NIR irradiation, suggesting that the intracellular ROS was mainly generated by the ICG released from the ID@MOS-Fc-CDHA nanocomposites via the NIR laser irradiation.

### 3.6. Synergistic Therapeutic Efficacy via the Chemo-, Photothermal, and Photodynamic Therapies

**Figure 7:**
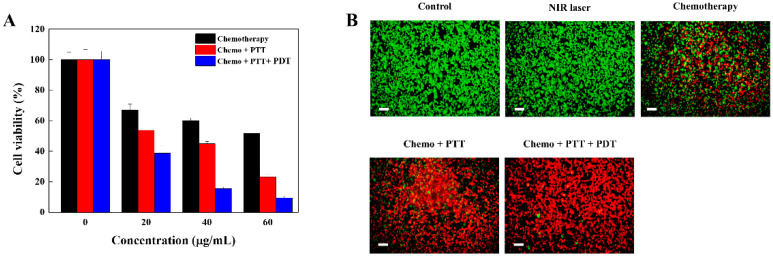
(**A**) Quantitative viability of the B16F10 cells treated with the ID@MOS-Fc-CDHA nanocomposites with or without the NIR laser irradiation (808 nm, 2 W/cm^2^, and 5 min) for the chemo-, photothermal, and photodynamic amulti-modal therapies. (**B**) Fluorescent images of the live/dead assay in the B16F10 cells after 60 μg/mL of the ID@MOS-Fc-CDHA nanocomposites with or without the NIR laser irradiation (808 nm, 2 W/cm^2^, and 5 min) for the synergistic therapeutic effects via the chemo-, photothermal, and photodynamic therapies. The cells were stained with calcein AM (live cells, green) and ethidium homodimer-1 (dead cells, red). Scale bars are 50 μm.

To investigate the therapeutic efficacy against the melanoma cells, via the multi-modal treatment (e.g., chemo-, photothermal, and photodynamic therapies), the B16F10 cells were cultured with different concentrations of the ID@MOS-Fc-CDHA nanocomposites (20~60 μg/mL) and the cytotoxicity was subsequently evaluated by a MTT assay (Figure 7A). As a chemotherapy, the B16F10 cells treated with nanocomposites were not exposed to the NIR laser irradiation and the viability of the B16F10 cells exhibited a dose-dependence on the nanocomposites. It was gradually decreased to 51%, because the Dox release was promoted by the microenvironment of the melanoma cells (e.g., low pH, HAase, high intracellular GSH, and H_2_O_2_) [2,32]. For the chemo-photothermal therapy, NaN_3_, as a ROS inhibitor, and the ID@MOS-Fc-CDHA nanocomposites were treated in the B16F10 cells following by a 808 nm NIR laser irradiation. The number of viable B16F10 cells decreased to 23% at the same concentration (60 μg/mL), indicating that about 28% of the B16F10 cells were dead by the photothermal therapy. Compared with the suppressed photodynamic therapeutic effect by NaN_3_, after the NIR irradiation in the B16F10 cells with the ID@MOS-Fc-CDHA nanocomposites, the percentage of cell viability decreased significantly, reaching approximately 9%. This potent anticancer efficacy was attributed to the chemo-, photothermal, and photodynamic treatments simultaneously by the intracellular antioxidant, enzyme, and H_2_O_2_ triggered and the melanoma targeted multi-functional nanocomposites. Furthermore, we conducted a live/dead assay to visualize the therapeutic efficacy of the melanoma cells via the NIR laser irradiation and nanocomposites (Figure 7B). In the control group, although a 808 nm NIR laser was irradiated to the B16F10 cells, a number of cells exhibited the green fluorescence, suggesting that only the NIR laser irradiation did not affect the cell viability [58]. In the B16F10 cells treated with the ID@MOS-Fc-CDHA nanocomposites without the NIR laser irradiation, some sporadic red fluorescence was observed, due to the chemo-therapeutic effect. Following the treatment of the NaN_3_, nanocomposites, and the NIR irradiation, a number of the B16F10 cells were changed to red fluorescence, indicating the combined chemo- and photothermal effects of the nanocomposites. Finally, when the ID@MOS-Fc-CDHA nanocomposites were treated to the B16F10 cells and exposed to the NIR laser, most B16F10 cells showed a strong red fluorescence and were dead by the chemo-, photothermal, and photodynamic therapies derived from the ID@MOS-Fc-CDHA nanocomposites, corresponding to the previous result of a MTT assay.

## 4. Conclusions

We designed the intracellular antioxidant, enzyme, and H_2_O_2_-triggered cancer cell targeted mesoporous organo-silica (MOS) nanocomposites to observe the therapeutic efficacy. By using the introduction of a novel CDHA gatekeeper and the specific interaction with the Fc molecule, the MOS-Fc-CDHA nanocomposites protected the drug leakage, controlled the drug release by the enzyme (HAase), and provided the selective internalization to the CD44-overexpressing cancer cells. Additionally, the MOS-Fc-CDHA nanocomposites could generate hyperthermia and cytotoxic ROS by a 808 nm NIR laser irradiation and regulate the Dox release by the presence of GSH, HAase, and H_2_O_2,_ due to the co-encapsulation of ICG and Dox. We observed that the ID@MOS-Fc-CDHA nanocomposites exhibited the excellent biocompatibility and the selective cellular uptake via the CD44-receptor mediated endocytosis. The ID@MOS-Fc-CDHA nanocomposites, under a 808 nm NIR laser irradiation also showed the potent therapeutic efficacy in the B16F10 melanoma cells by the synergistic chemo-, photothermal, and photodynamic therapies. Therefore, this antioxidant, enzyme, and H_2_O_2_-triggered nanocomposite could be a potentially powerful carrier for the multi-modal therapy applications.

## Data Availability

The data presented in the study are available in this manuscript.

## Supplementary Materials

The following supporting information can be downloaded at: https://www.mdpi.com/article/10.3390/antiox11112137/s1, Figure S1: Size distribution of the MOS, MOS-Fc, and MOS-Fc-CDHA nanocomposites, Scheme S1: Synthesis process of the melanoma targeted and stimuli-responsive CDHA gatekeeper, Figure S2: ^1^H NMR spectra of *β*-CD-NH_2_, HA, and the CDHA gatekeeper, Figure S3: FTIR spectra of the MOS, MOS-Fc, and MOS-Fc-CDHA nanocomposites. Figure S4: UV-vis spectra of free ICG, free Dox, and the ID@MOS-Fc-CDHA nanocomposites, Figure S5: Cytotoxicity analysis of the MOS-Fc-CDHA nanocomposites against the NIH-3T3 fibroblast and the B16F10 melanoma cells.
