# Supplementary material for: Antioxidant, Enzyme, and H2O2-Triggered Melanoma Targeted Mesoporous Organo-Silica Nanocomposites for Synergistic Cancer Therapy
Source: Antioxidants (Basel). 2022 Oct 28;11(11):2137. doi: 10.3390/antiox11112137 (PMC9686543; doi:10.3390/antiox11112137)
Supplement: Supplementary file 1 [file antioxidants-11-02137-s001.zip › antioxidants-1981392-supplementary.pdf]

SUPPLEMENTARY MATERIALS

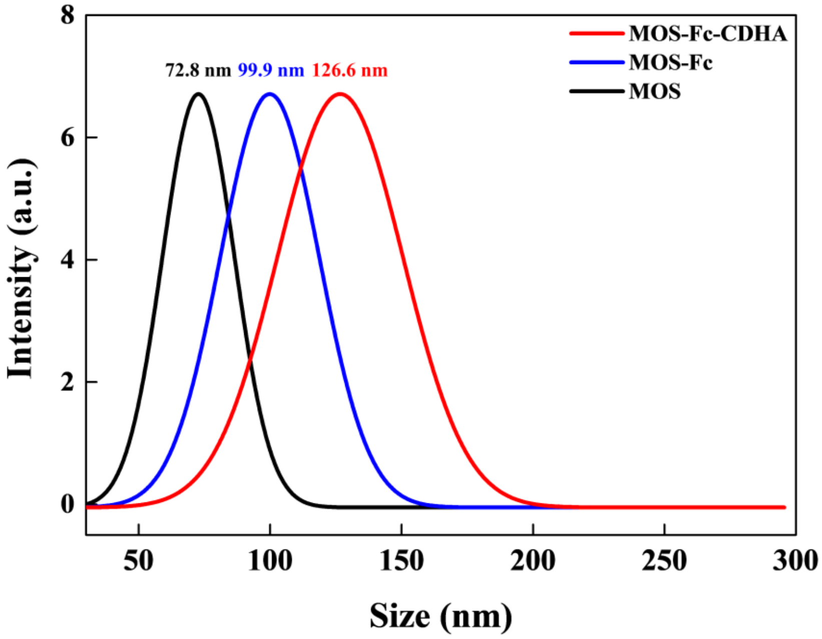

Figure S1. Size distribution of MOS, MOS-Fc, and MOS-Fc-CDHA nanocomposites

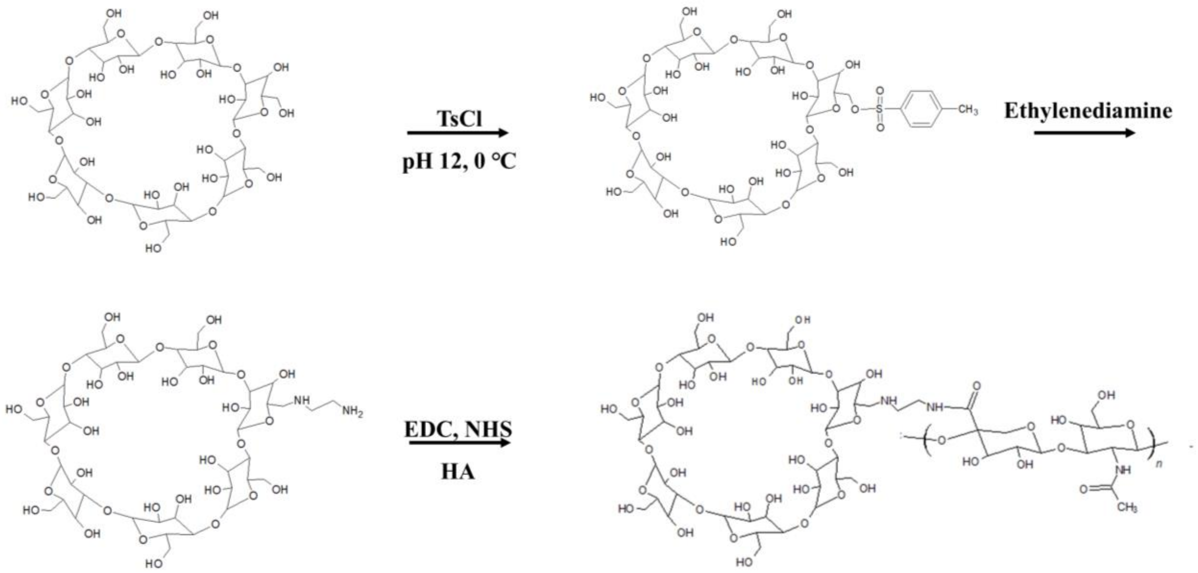

Scheme S1. Synthesis proecess of melanoma targeted and stimuli-responsive CDHA gatekeeper

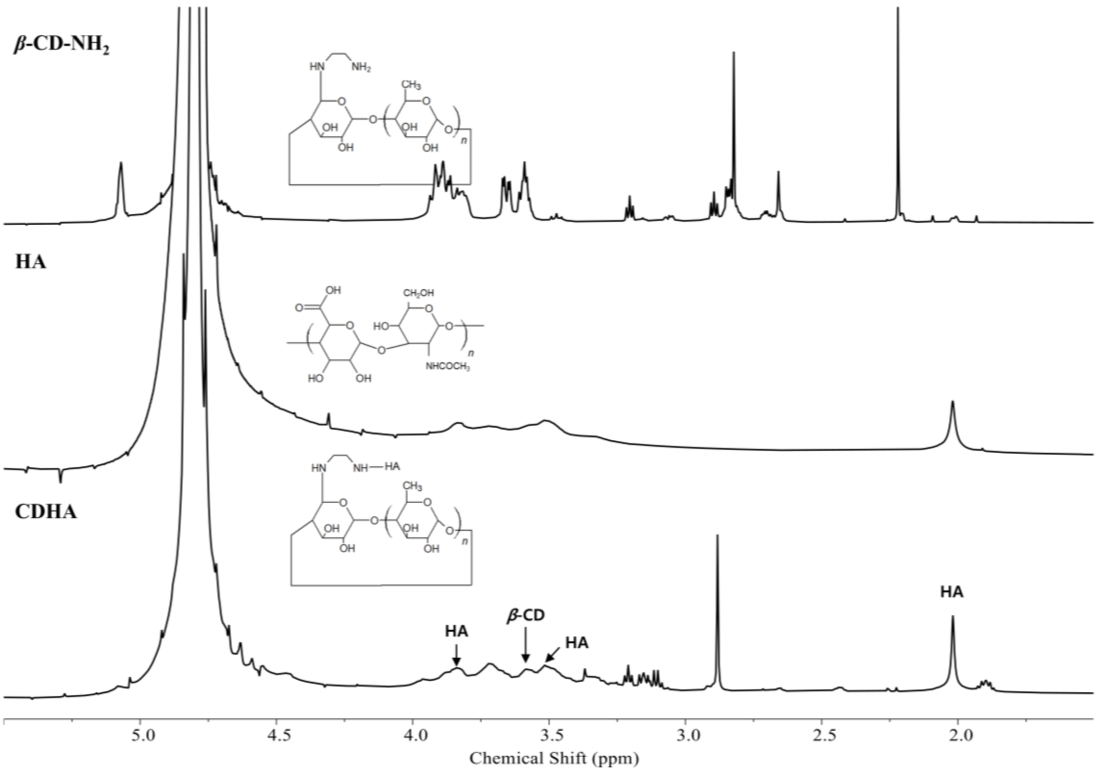

Figure S2. ^{1}H NMR spectra of β-CD-NH_2, HA, and CDHA gatekeeper

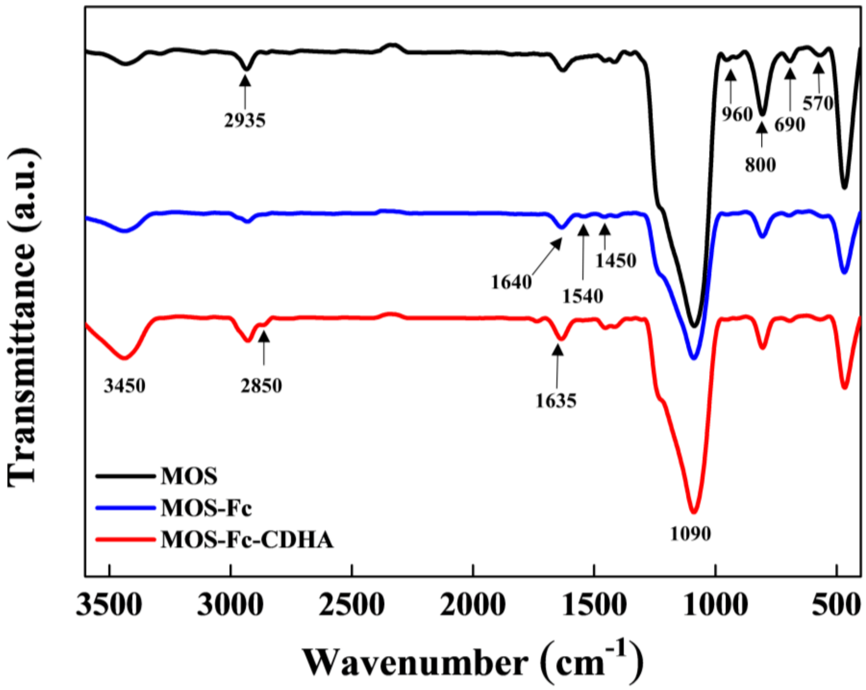

Figure S3. FTIR spectra of MOS, MOS-Fc, and MOS-Fc-CDHA nanocomposites.

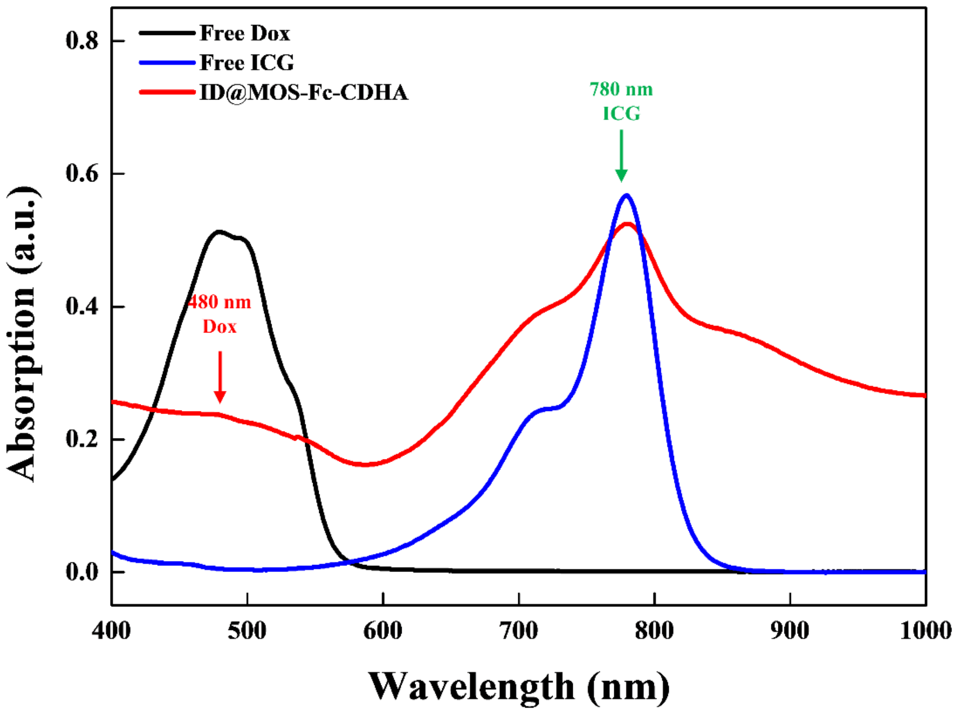

Figure S4. UV-vis spectra of free ICG, free Dox, and ID@MOS-Fc-CDHA nanocomposites.

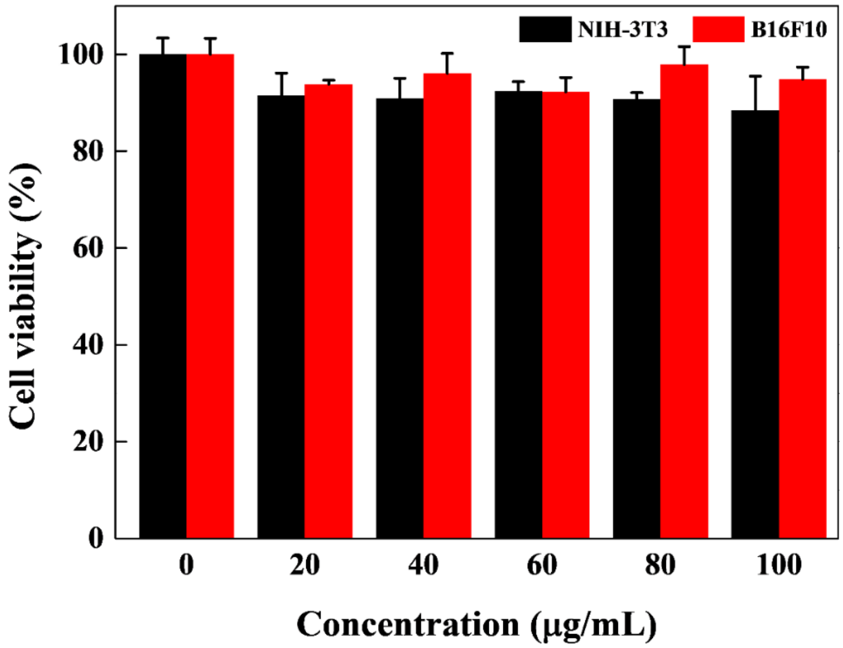

Figure S5. Cytotocity analysis of MOS-Fc-CDHA nanocomposites against NIH-3T3 fibroblast and B16F10 melanoma cells.
